# The implementation of neo- and nonbinary pronouns: a review of current research and future challenges

**DOI:** 10.3389/fpsyg.2024.1507858

**Published:** 2025-01-30

**Authors:** Emma A. Renström

**Affiliations:** Kristianstad University, Kristianstad, Sweden

**Keywords:** pronouns, attitudes, gender conceptualization, neopronouns, nonbinary pronouns, gender-inclusive language, politicization

## Abstract

This review explores the current state of research on attitudes toward and the use of neo- and nonbinary pronouns, as well as their effects on gender conceptualization. Due to the limited scope of existing studies, this review focuses on Swedish and English. Additionally, I will examine resistance to gender-inclusive language and linguistic gender reforms, with a particular emphasis on nonbinary pronouns and the politicization of such reforms, which represents a significant barrier to the adoption of gender-inclusive language. More research is needed to explore attitudes toward, usage of, and the consequences of neo- and nonbinary pronouns across a wide range of languages. Moreover, it is crucial to investigate the politicized polarization surrounding these reforms to better understand when and why people (do not) use nonbinary pronouns and the broader implications of these pronouns for gender conceptualization in the future. This review is structured as follows: I will first describe the general function of pronouns and discuss the interplay between language, gender, and cognition in relation to pronouns. Then, I will examine the implementation of gender-inclusive pronouns in Swedish and English, the dual nature of many gender-inclusive pronouns, and how this duality influences attitudes and usage. I conclude by discussing future research venues that I see, mainly connected to better understanding the politicization of gender-inclusive language and how this politicization and polarization influences attitudes to and use of nonbinary pronouns and effects of nonbinary pronouns in gender conceptualization.

## Introduction

Language is a tool for transferring cultural knowledge, for example about gender (Beukeboom and Burgers, 2019). Research shows that language influences cognitive processes (Lucy, 1992; Samuel et al., 2019; Fiedler, 2008). Words can influence how events in the world and other people are perceived, leading to initiatives to create a more inclusive language. In line with this, words activate mental representations and evaluations both implicitly and explicitly, and hence, word choices can trigger exclusion, stereotypes, discrimination, and harassment related to gender (Sczesny et al., 2016). Consequently, the research field of gender-fair language is dedicated to better understanding how language can be used to create linguistic inclusion and visibility instead of exclusion.

In line with the idea to make language more inclusive, nonbinary pronouns constitute a relatively recent linguistic development that aims to make language more inclusive by adding a third personal pronoun singular to languages that already have pronouns representing women and men (e.g., English, French, and Dutch). Nonbinary pronouns are sometimes also neopronouns—newly invented words. However, these pronouns are sometimes revamped versions of older pronouns, such as singular *they* in English. Hence, even though referred to as nonbinary, these pronouns often have a dual meaning: a *nonbinary* meaning, referring to individuals with nonbinary gender identities, and a *generic* meaning, referring to anyone regardless of gender (Renström et al., 2022a).

Swedish was the first language to add a nonbinary pronoun, *hen*, to the official dictionary (SAOL, 2015). Since then, other languages have followed suit. In English, singular *they* is the most common nonbinary pronoun known to the majority of English speakers. Another alternative is *ze*, which is relatively unknown (Lindqvist et al., 2019; Renström et al., 2023a). In French, there is a recent initiative to add the nonbinary pronoun *iel* (Wagener, 2021). In Danish, *hen* and singular *de* (they) are suggested, and in Dutch, both *hen* and *die* are proposed (DeCock et al., 2024; Hjorth-Nebel Miltersen et al., 2022). While these are examples of a global trend, this review will focus on Swedish and English due to a lack of published research (in English) on attitudes, use, and consequences of nonbinary pronouns in other languages.

Common to many of these initiatives is a strong resistance against them as they challenge societal structures and deeply rooted identities related to the view of gender as a binary construct (Renström and Klysing, 2024; Hekanaho, 2020; Morgenroth et al., 2020). The politicization of nonbinary pronouns likely influences how the public receives them in terms of attitudes and use, but also how they influence the construction of gender—how the concept of gender is perceived as (non)binary.

The predominant view of gender constructs it as a binary social category derived from two biological sexes, which are generally seen as two mutually exclusive categories (Hyde et al., 2019; Morgenroth and Ryan, 2018). Both feminists and sexual minority individuals have challenged this view in different ways, including through gender-inclusive language (Gustafsson Sendén et al., 2015, 2021).

Third-person pronouns have become important markers of gender-ideological positions by their relation to gender identity, including the potential for signaling to have a gender identity that defies a binary gender system (Hekanaho, 2020, 2022). Besides expanding gender categories, some nonbinary pronouns can also function to de-emphasize gender in language by removing gender information altogether. A better term for such pronouns would be gender-inclusive pronouns. Gender-inclusive pronouns often serve two functions: to emphasize the existence of multiple gender identities or to decrease the overall salience of gender in language.

The binary conceptualization of gender functions prescriptively to specify what is desired of women and men and by proscriptively specifying what is not accepted (Morgenroth et al., 2020; Prentice and Carranza, 2002). Individuals who violate these expectations are socially punished (Rudman et al., 2012), which contributes to the marginalization of sexual and gender minority groups (Thoma et al., 2021). Two strategies have been suggested to challenge the binary conceptualization of gender and battle such problematic effects (Morgenroth and Ryan, 2020). *De-gendering strategies* aim to remove or minimize the salience of gender altogether, while *multi-gendering strategies* aim to draw attention to the fact that gender is not binary. These two strategies correspond to the two ways in which many gender-inclusive pronouns, such as singular *they*, are used. The active strategy type is defined by the context in which the gender-inclusive pronoun occurs (Renström et al., 2022a). When a gender-inclusive pronoun is used to anonymize or to replace the paired pronoun form *he/she* in generic texts, it functions to decrease the influence of gender, but when a gender-inclusive pronoun is used to refer to individuals with nonbinary gender identities, it serves to increase the salience of other gender identities than the binary woman/man.

Gender-inclusive language reforms, including the use of gender-inclusive pronouns, face resistance (Blaubergs, 1980; Parks and Roberton, 1998; Bradley, 2020; Bradley et al., 2019; Hekanaho, 2020; Vergoossen et al., 2020a). This resistance is largely grounded in gender-ideological convictions about gender being an essential and binary category and a desire to keep gendered power structures intact (Douglas and Sutton, 2014; Parks and Roberton, 2005; Hekanaho, 2022). However, as de-gendering and multi-gendering strategies to some extent challenge core convictions of different ideologies, the origins of resistance against gender-inclusive pronouns may also differ depending on which strategy is salient (Morgenroth et al., 2020; Renström et al., 2022a), which poses a challenge to the study of neo- and nonbinary pronouns.

## Neo- and nonbinary pronouns

In this article, I use the terms neopronouns and nonbinary pronouns, but what terms to use is not straightforward. In fact, when referring to pronouns that have a dual function, such as singular *they*, nonbinary is not correct since a nonbinary pronoun should specifically refer to a pronoun used for individuals with nonbinary gender identity in the same way that *she* should be used about individuals identifying as women.

The definition of a neopronoun is that it is new. It is a word that did not exist in the language before and has been created with a specific meaning. The definition of a nonbinary pronoun is that it applies to individuals with nonbinary gender identities. Nonbinary refers to individuals who do not define themselves as either of the traditional binary genders, woman or man. Hence, a nonbinary pronoun may or may not be a neopronoun. The most illustrative example of this is the use of singular *they* as a nonbinary pronoun. However, singular *they* has a long history of being used to avoid gender cues or when the gender of a referent is unknown, and thus, singular *they* cannot be considered a neopronoun. Because singular *they* is used differently, it is not entirely correct to call singular *they* a nonbinary pronoun either. Examples of nonbinary neopronouns in English are, for instance, *ze* or *xe*, which should be considered both neopronouns and nonbinary pronouns.

Another example is the Swedish pronoun *hen*, which is new and nonbinary. However, *hen* specifically has two meanings—the nonbinary meaning and the generic meaning. Again, due to the dual meaning, it is not entirely correct to label *hen* a nonbinary pronoun. Hence, another term that would fit better for both *hen* and singular *they* is *gender-inclusive,* meaning the inclusion of all genders regardless of what they may be. The same is true for the Danish initiative singular *de* (they) (Hjort Miltersen, 2020).

Other terms that are used to refer to these kinds of pronouns are gender-neutral or gender-fair pronouns. The idea behind the term “neutral” is that the removal of gender cues should make perceivers less biased because there is no linguistically gendered cue to prime cognitive processing. However, neutralizing runs the risk of assumptions that all genders are equal in every sense. While it is true that, for instance, cognitive abilities do not greatly differ between women and men (Hyde et al., 2019), the experiences of being a woman, a man, or a transperson differ greatly, which impacts behavior. This relates to how gender stereotypes foster gendered expectations and behavior. Hence, gender-neutral language reforms should be used and implemented with caution.

In the social psychological literature on language and gender, the term *gender-fair* language is often used. While gender-fair language theoretically aims to create broader gender categories that could encompass individuals of any gender (Sczesny et al., 2016), in practice, this research field has mainly been devoted to promoting fairness for women relative to men. However, the field is evolving, with more scholars attempting to adopt a broader perspective on gender in language. Within this literature, two principal strategies for achieving gender-fair language are frequently discussed: neutralization and balancing (also referred to as feminization).

However, as mentioned, neutralization might not be beneficial in the long run, and balancing implies the linguistic representation of different genders, which mainly has been women—neither of these strategies is, in fact, fair to all genders. The unfairness of balancing becomes very clear with the example of how pronoun use has evolved from a generic *he* to the balanced (“gender-fair”) paired form *he/she*. It is safe to say that *he/she* does not represent all genders and reinforces gender binarity (Bigler and Leaper, 2015; Lindqvist et al., 2019; Renström et al., 2023a). Hence, balancing, as it has been used in the binary sense, is not gender-fair.

Again, the term I suggest to better encompass what gender-fair language aims to achieve is *gender-inclusive*. Hence, I will use *gender-inclusive pronouns* when referring to both neopronouns and nonbinary pronouns when they aim to include all genders. It should be noted that this is not always the case. This is the case for the English pronoun singular *they*, which can be used to include all genders. However, regarding specifically nonbinary pronouns such as English *xe*, this is not the case—*xe* is supposed to be used for individuals with nonbinary gender identities in the same sense that *she* is assumed to be used for women and *he* for men.

### The implementation of neo- and nonbinary pronouns

Swedish was the first language to officially implement a third pronoun (Bäck et al., 2018) in the Swedish Academy Glossary, constituting a non-official guide to the Swedish language (SAOL, 2015). However, *hen* was mentioned in 1966 in a newspaper and suggested by the linguist Rolf Dunås as a replacement for the paired form *he/she*. Dunås was inspired by the Finnish language that does not have pronouns representing women and men (i.e., *she* and *he*), but only a gender-inclusive pronoun, *hän* (Ledin and Lyngfelt, 2013). However, at this time, *hen* did not have any noticeable impact. Instead, in the early 2010s, *hen* started being used as a nonbinary pronoun in some LGBTQIA communities. The public breakthrough for *hen* came in 2012 when a children’s book was published where the main character was referred to as *hen* and a debate article was written by the book’s publishers and a feminist linguist advocating for *hen* as a gender-inclusive pronoun. This action was coordinated and cleverly focused on children, guaranteeing an impactful public debate. The debate article discussed that gendered pronouns hinder children, and by using *hen* and gender-neutral language, all children could identify with the main character (Milles, 2013). This coordinated action sparked a heated, polarized, and politicized debate about the nature of gender and the rights of parents and children. While the debate was quite negative, it made all Swedish speakers aware of the pronoun *hen* (Gustafsson Sendén et al., 2015).

Not all neo- and nonbinary pronouns are implemented in this way. When it comes to singular *they*, there was mainly a change in how the pronoun was used, which subsequently led to a change in the Merriam-Webster dictionary regarding the definition of singular *they*. Singular *they* is traced back to the late 1300s when it appeared in sentences replacing *he* or *she* (Oxford English Dictionary).^1^ However, its use as specifically referring to individuals with nonbinary gender identities is relatively new. For instance, Merriam-Webster appointed *they* as Word of the Year in 2015, which gained quite a lot of attention and helped bring awareness to the word. In 2022, almost all American English-speaking participants in a study on singular *they* knew that the word could be used nonbinary (Renström et al., 2023a).

Singular *they* is the most well-known gender-inclusive pronoun in English. Singular *they* has been a long-standing candidate in English for expressing gender neutrality (Balhorn, 2004). Before, some guides (e.g., Strunk and White, 1972) suggested that *he* should be used generically and argued against singular *they* due to it being a plural form, which by default cannot refer to a single entity. Generic *he* was opposed based on arguments that it is associated with masculinity and men, even though it is supposed to be neutral (Moulton et al., 1978).

There are many more initiatives in English, such as *ze* and *xe,* to mention a few. However, these pronouns have not received the same public attention and are relatively unknown. In 2022, about 25% of American English speakers knew that *ze* was a nonbinary pronoun (Renström et al., 2023a). Similarly, a Dutch study recently found that only half of the participants had knowledge about gender-inclusive pronouns in Dutch (DeCock et al., 2024). This is problematic because if people are unaware of the use of a pronoun, they are unlikely to use it, and it may also influence how a text with the pronoun is processed. Hekanaho (2020), for instance, found that when encountering *ze*, some people simply thought it was a typo, and it should be *he*.

Renström et al. (2024) have explored general attitudes to *hen* as a “gender-neutral” pronoun in a series of studies. As expected, participants were quite negative in the early studies of 2012 and 2015 (Gustafsson Sendén et al., 2015). In one study analyzing changes in attitudes between 2015 and 2018, Gustafsson Sendén et al. (2021) found that time was the strongest predictor of attitudes, even when controlling for several other important factors. They observed the main change among younger people. In a follow-up study in 2021, Renström et al. (2024) found that the change from 2018 to 2021 is smaller, but this time, the change mainly lies with middle-aged and older people. This data suggests that time has the potential to influence attitudes in a longer perspective, and people become more positive or at least less negative (Renström et al., 2024). However, one caveat with this data is that there is no separation between the different meanings of *hen*—that is, when participants answered the questions about their attitudes to *hen*, we did not know if they were thinking about *hen* as nonbinary or as a generic pronoun replacing *he/she*. This distinction is consequential because there may be different origins of resistance against gender-inclusive pronouns depending on how their meaning is perceived (Renström et al., 2022a).

## Language and cognition

Languages are dynamic constructs, constantly changing to accommodate variations in the world and how people perceive objects and events. However, although language reflects how the world is constructed, language also *influences* the construction of the world (Fiedler, 2008; Lucy, 1992). This means that language can modify how individuals perceive objects and other people—a phenomenon known as the Whorfian hypothesis or the linguistic relativity hypothesis (Whorf, 1956). This idea is also captured in the term conceptual engineering—the striving for change in linguistic practices by, for instance, the use of novel words (Koch and Lupyan, 2024).

The linguistic relativity hypothesis states that language influences how the world is conceptualized (Samuel et al., 2019; Whorf, 1956). In its original form, this hypothesis claimed that language could completely change concepts, but a softer version - with more empirical support - claimed that language should be seen as a way to influence perceptions (Lucy, 1992). For example, a definite article defining the feminine/masculine gender of a certain noun appears to influence how that noun is perceived. In one study, bilingual participants took part in an experiment conducted in English and were asked to describe a bridge. Because the experiment was performed in English, no grammatical gender cues were present. Yet German-speaking participants used mainly stereotypically feminine terms to describe a bridge (e.g., slender, elegant) while Spanish-speaking participants used mainly stereotypically masculine terms (e.g., big, sturdy). The conclusion was that because the noun bridge is feminine in German (*Die brücke*) but masculine in Spanish (*el puente*), grammatical gender bias perceptions of non-gendered objects (Boroditsky et al., 2003). Consequently, it can be assumed that, in general, German and Spanish-speaking individuals have different prototypes of the noun bridge.

A prototype can be defined as an exemplar of a category that best represents the category and is contingent upon individual experiences and culture. Different exemplars can have varying degrees of belonging to a conceptual category, making some exemplars more representative than others (Rosch, 1973). In this sense, we can assume that in most languages, people have clear prototypes of the concept of gender that include normative cis-women and -men with a certain appearance and having certain qualities. Even though such prototypes, or the concept of gender, may vary as a function of context (for a discussion, see, for instance, Mazzuca et al., 2020, 2024), the predominant view of gender in Western countries is binary. When the prototype is activated, exemplars that fit better with this prototype will likely be more positively evaluated than exemplars with a worse fit. Research shows that when women and men display gender-stereotype congruent behavior, they are more positively evaluated than when the stereotype and behavior is incongruent (Eagly and Karau, 2002).

More recently, a study using natural language processing showed a similar connection between the grammatical gender of an inanimate noun and the verbs and adjectives associated with that noun (Williams et al., 2021). These studies show that language influences human conceptualization (Flaherty, 2001) and that gender cues in language have the power to influence how gender is constructed. Following the linguistic relativity hypothesis, a substantial change in how a concept (e.g., gender) is grammatically defined (e.g., through personal pronouns) should influence that conceptualization (e.g., the construction of gender) (see also Borghi and Mazzuca, 2023 for a discussion on properties of a concept that facilitate linguistic relativity).

It should be noted that some studies have not found the expected link between grammatical gender in language and conceptualization (e.g., Elpers et al., 2022), and some scholars question whether grammatical gender is a useful tool to investigate linguistic relativity (Samuel et al., 2019). Regardless, research on the negative consequences of gender cues in language is vast. Gender is more or less grammatically salient in different languages. Grammatical language structures can be categorized by how nouns and pronouns are gendered regarding feminine and masculine grammatical gender (Gygax et al., 2019; Prewitt-Freilino et al., 2012). Three language groups have been categorized: languages with natural gender where pronouns but not nouns are gendered (e.g., English, Norwegian); gendered languages where both nouns and pronouns are gendered (e.g., French, Russian); and genderless languages where neither nouns nor pronouns are gendered (e.g., Finnish, Turkish) (Prewitt-Freilino et al., 2012; Siewierska, 2013; Stahlberg et al., 2007).

Nouns and pronouns with feminine/masculine grammatical genders activate gender categorization, stereotypes, and prejudice (Bigler and Leaper, 2015; Lindqvist et al., 2019). The grammatical structure of languages relates to prejudice against women, sexism, and national-level gender equality (DeFranza et al., 2020; Prewitt-Freilino et al., 2012). For example, the more the grammar system in a language distinguishes between feminine and masculine gender, the fewer women are represented in business power positions (Santacreu-Vasut et al., 2014) and the larger the wage gaps between women and men are (Shoham and Lee, 2018).

In many grammatically gendered languages, the generic and plural form of social roles is often masculine (although exceptions exist). Some roles and nouns have suffixes indicative of men and masculinity, such as chair*man*, but also hu*man*. These suffixes provide linguistic gender cues, which are associated with men (McConnell and Fazio, 1996). Note that there are exceptions such as female suffixes for role nouns as well, for example, mid*wife* in English, but also *sjuksyster* (Swedish) or *Krankenswester* (German), both of the latter meaning “sister for the sick,” which is the word for a nurse. Regardless of whether the suffix is masculine or feminine, such suffixes indicate that one gender is better suited than another for the role, which biases perceivers and perpetuates gender stereotypes.

Furthermore, it is more common with masculine suffixes for roles and nouns that are not stereotypically associated with men or women, such as chair*man* or ombuds*man*. Using such masculine generics influences cognitive processing and bias retrieval of masculine exemplars to a greater extent than feminine exemplars, resulting in a male bias (Hellinger and Bussman, 2002; Stahlberg et al., 2007). In such cases, the male bias is evoked due to linguistic cues associated with men.

A male bias is also observed when non-gendered words are associated with masculinity (Lindqvist et al., 2019; Liu et al., 2018). For example, an undefined person is often perceived as a man (Bailey and LaFrance, 2017; Bem, 1993; Hegarty and Buechel, 2006). This bias has been found for linguistically neutral words (e.g., the word “the applicant”) that do not carry any grammatical or semantical gender cues (Lindqvist et al., 2019). That such neutral terms are associated with masculinity exemplifies an androcentric worldview where men constitute the norm (Eagly and Kite, 1987). There is also a whiteness bias, meaning that the perception of an undefined person is that of a White person (Bailey and LaFrance, 2017). Moreover, there is a heteronormative bias where the perception of an undefined woman or man is that of a heterosexual woman or man (Klysing, 2023). Together, these biases in supposedly “neutral” words imply that the mental associations of non-defined categories are filled by the norm.

Even in languages without gendered pronouns, such as Finnish and Turkish, their respective pronouns have a male bias (Renström et al., 2023b). In a recent article, general descriptions of an individual in Finnish or Turkish referred to as *hän* (i.e., the Finnish third-person singular pronoun) or *o* (i.e., the Turkish third-person singular pronoun) resulted in more associations with a man than a woman. It is interesting that this was the case in both countries, which vary greatly in national-level gender equality (as assessed by the World Economic Forum, 2021). One conclusion is that androcentrism is deeply rooted in the human mind and possibly associated with a patriarchal culture (Renström et al., 2023b).

Given that most Western societies are patriarchal, there also exists a “male as norm” effect, which shows in varying ways in language and perpetuates the precedence of men and masculinity over other gender identities. Men and masculine terms are often more prevalent than women, and feminine terms and men are presented first. For instance, the paired pronoun form *he/she* presents the masculine form before the feminine form. Such practices lead to perceptions of men as the norm and women as the deviation from the norm. This matters in hiring processes, for instance. If the norm is masculine and perceivers have a man in mind for a position, a woman will be more negatively evaluated, while a man appears more suitable because he constitutes a match. For instance, when masculine generic terms are used in job advertisements, women become less motivated to apply because they do not feel targeted (Bem and Bem, 1973; Gaucher et al., 2011). Thus, androcentrism, not only in language, influences the demand side of hiring processes by influencing who is suitable for a position but also influences who will apply for the position, that is, the supply side. Hence, language is consequential on both micro and macro levels.

In sum, gender cues in language, whether grammatical, linguistic, or based on the order of presentation, tend to favor men over women or other genders and often function to increase binary gender stereotypes about women and men. Hence, gender in language is problematic, which has long been noted by women’s activists and gender-interested scholars from varying disciplines.

### Decreasing the negative consequences of gender in language

In psychology, gender-fair language describes a research field dedicated to resolving how language can be used to increase equality and inclusion—that is, to activate broader, rather than narrower, category boundaries. Previously, gender-fair language initiatives have mainly focused on decreasing linguistic androcentrism (i.e., the conflation of humanity with masculinity) by either increasing the linguistic salience of femininity or neutralizing the salience of gender in language altogether (Sczesny et al., 2016). Which strategy that has been predominant in a language is mainly contingent upon the grammatical structure. In languages with gendered nouns and pronouns (i.e., gendered languages), the reform balancing or feminization has dominated. The background is found in the widespread use of masculine generics, and research shows that such forms are male-biased. Hence, increasing women’s visibility has been an important step in these languages. In languages with gendered pronouns but not nouns (i.e., natural gendered languages), a neutralization strategy is more often employed, implying the removal of gendered information in language.

To increase linguistic salience of femininity, feminine word forms can be included in addition to masculine generics, such as the use of *he/she* instead of generic *he* or including feminine forms in occupational titles (e.g., *Lehrer/Lehrerinnen*, meaning male/female teacher in German). Such reforms make women linguistically visible (Sczesny et al., 2016). However, pairing feminine/masculine words can reinforce the notion of gender/sex as a binary construct (Butler, 1988; Lindqvist et al., 2020; Morgenroth et al., 2020) when the paired forms are presented as a unit highlighting only two genders. Hence, individuals with intersex variations and/or nonbinary gender identities become invisible (Hyde et al., 2019; Lindqvist et al., 2020).

While balancing strategies aim to visualize feminine gender, neutralization aims to decrease the influence of gender on cognition by reducing the frequency of gendered words in a language. For example, using *chair* instead of *chairman* activates broader and more gender-inclusive characteristics associated with that position. Other examples are to use *firefighters* instead of *firemen* or *police officers* instead of *policemen*. As expected, avoiding masculine generics in professional titles and job adverts makes women more interested in applying for that position (Sczesny et al., 2016).

These strategies, however, pose some problems. First, balancing (or feminization) is obviously not balancing in an all-encompassing sense since it refers to the balancing of feminine and masculine forms. This strategy thus diminishes the visibility of other gender identities. Moreover, balancing feminine and masculine forms by presenting them as two parts of a whole—two mutually exclusive categories—reinforces a binary view of gender (Lindqvist et al., 2020) and binary gender stereotypes about women and men (Bigler and Leaper, 2015). Another problem with this strategy is order effects. When a word pair is presented, the word that is presented first is often processed as the dominating, more important, or hierarchically higher one (Hegarty et al., 2016). When word pairs are presented, they are most often presented with the masculine form first (e.g., *he/she*, or in the case of role nouns, *studenten/studentinnen* in German), although there are exceptions such as *ladies and gentlemen*. Gabriel and Gygax (2008) found that the word presented first was given more attention than the word presented second. When the word businesswoman was presented before the word businessman, the woman-form was seen as more central than the man-form (Kesebir, 2017). However, this presentation format with the woman-form first is less common.

Regarding neutralization, role forms are often not gendered but neutral. However, this is a modification of the truth because in many of these languages, such as Norwegian, the historical masculine form is the form most often used (Swan, 1992). In such languages, the feminine form has more or less been completely dropped from the language. While not considered a masculine generic in that the masculine form is used generically simultaneously with a feminine form existing, it is nevertheless a masculine form. When role names are not clearly stereotypically gendered, such as nurse or pilot, the historically and grammatically masculine forms are male biased (Gabriel and Gygax, 2008). This means that when neutralized forms that most often are historically masculine are used, people tend to associate these with men and masculinity unless the neutralized form is heavily stereotypical.

Both strategies proposed to make the language more inclusive and ensure the visibility of different genders have notable flaws. In a review article, Gabriel et al. (2018) discussed these two strategies. Modifying gendered role nouns, such as replacing gendered suffixes with neutral ones, is relatively straightforward—at least from the perspective of language production (Gabriel et al., 2018). For instance, changing *fireman* to *firefighter* poses no significant linguistic or grammatical challenges. However, resistance may arise due to ideological views. In contrast, for languages with more grammaticalized gender, implementing such changes is more complex, as it requires altering entire sentence structures.

### Toward a new terminology: de-gendering and multi-gendering strategies

Instead of the terms balancing/feminization and neutralization, which are closely connected to linguistic reforms, I use the broader terms proposed by Morgenroth et al. (2020) and Morgenroth and Ryan (2020)—*de-gendering* and *multi-gendering*. These strategies aim to counter the general binary conceptualization of gender and are not limited to language. De-gendering strategies aim to decrease the influence of gender by removing gender cues. While not limited to linguistic reforms, when applied to language, this practice equates to neutralization. Multi-gendering strategies, such as balancing in language, aim to emphasize the diversity of gender identities but explicitly include identities beyond the binary genders. While balancing has traditionally been used to increase the visibility of women in language, multi-gendering strategies explicitly incorporate nonbinary identities into linguistic practices.

To illustrate how de-gendering and multi-gendering strategies can be implemented, Klysing et al. (2021) showed organization descriptions to participants in an experiment, where the descriptions contained different equal employment opportunity statements, which either emphasized binary gender, gender as diverse (multi-gender), or gender as irrelevant (de-gender). In a control condition, participants were not shown any statement. The results showed that gender minority participants felt more secure in organizations that used either a de-gendering or multi-gendering statement, which increased organizational attractiveness. The type of statement had no effect on gender majority participants. These results clearly illustrate the consequentiality of both de-gendering and multi-gendering strategies.

As previously mentioned, gender-inclusive pronouns, such as neo- and nonbinary pronouns, may carry a dual meaning. Depending on the context in which they are used, these pronouns can align with either de-gendering or multi-gendering strategies (Renström et al., 2022a). Before exploring the dual meaning of such pronouns and consequences, I will first discuss the relationship between pronouns and the construction of gender as a binary or nonbinary concept.

## Pronouns and the construction of gender

Gender (sometimes referred to as sex/gender) is most often constructed as a binary concept. The binary conceptualization of gender prescribes appropriate and proscribes inappropriate traits and behaviors for women and men (Morgenroth et al., 2020; Prentice and Carranza, 2002; Renström et al., 2023a). When these expectations are violated, individuals are often socially sanctioned, which explains the marginalization of gender minority groups (Thoma et al., 2021) but also contributes to the stereotyping of cis-gender women and men (Rudman et al., 2012). Thus, decreasing the binary conceptualization is imperative.

Pronouns denote gender identity. Although *she* and *he* are not synonymous with feminine or masculine gender identities, pronouns have increasingly become markers of gender identity (Hekanaho, 2020). In everyday language, pronouns facilitate communication by allowing stereotypical inferences about individuals when pronouns representing women and men are used (i.e., *she* and *he*). If a person is referred to as *he*, social perceivers assume that this target is a man and should perform stereotypically masculine tasks and roles and have corresponding attitudes (Eagly, 1987). When meeting or hearing about a new person, an impression is formed that provides a mental framework for understanding the target’s behavior (Riggio and Friedman, 1986). By using the available information, such as pronouns or gender, social perceivers can effectively categorize targets into meaningful categories. While such inferences are based on stereotypes and thus should not be assumed to be valid for every category member, they facilitate everyday interactions and communications by decreasing cognitive load (Macrae et al., 1994). This implies that the use of gendered pronouns influences social perception, which may have far-reaching consequences. However, what happens when gender-inclusive pronouns, such as neopronouns or nonbinary pronouns, are used? How does such use influence social perceivers and the construction of gender?

In one experiment, Lindqvist et al. (2019) tested to what extent different labels, including the paired pronoun form *he/she* and the gender-inclusive pronoun *hen* in Swedish, were associated with a male bias. Participants were shown a description of a candidate for a job as a real estate agent (which is gender balanced when it comes to women and men, according to Swedish official statistics), ostensibly written by a professional recruiter. The candidate was referred to using the labels *the applicant*, *NN* (short for Latin Nomen Nescio, translating to “do not know the name,” which is sometimes used to anonymize), *he/she,* or *hen*. Participants were then asked to indicate who they thought they had read about by selecting one out of several photos showing cis-gender women and men. The results showed that both *the applicant* and *NN* were male-biased, with participants selecting photos of men to a significantly larger degree than what would have been expected had they selected randomly. However, both *he/she* and *hen* led to a more even distribution of selected women and men.

Moreover, the experiment was replicated in English using *the applicant*, the paired form *he/she*, the nonbinary neopronoun *ze,* and the gender-inclusive pronoun singular *they*. The results showed that *the applicant* was again male-biased, but singular *they* was also male-biased. While singular *they* has been quite successfully launched as a gender-inclusive, nonbinary pronoun, social perceivers categorize singular *they* as a man. *he/she* and *ze* resulted in an even distribution of women and men targets, thus not being male biased. One difficulty with *ze* is that many participants had not heard about *ze* as a nonbinary pronoun. Hekanaho (2020) found that some people may understand *ze* as a misspelled *he*. That appears unlikely in this case since that should have led participants to select a photo of a man.

Nevertheless, the fact that many participants (approximately 60% in this study) had no knowledge about *ze* as a pronoun cannot be disregarded, and we do not know how this influences social perceivers’ gender conceptualization. These results indicate that neutral terms are associated with men and masculinity, even without grammatical or linguistic cues. This could be based on a patriarchal and androcentric worldview. Hence, when social perceivers hear about a person referred to using a non-gendered term in a neutral context, they infer masculinity (Gabriel and Gygax, 2008).

While *he/she* was not associated with a male bias, there are problems using paired pronouns as discussed above, such as order effects, the presentation of a coherent unit consisting of two mutually exclusive categories, and stereotypes associated with women and men. To explore the consequences of using the paired form *he/she* on gender conceptualization, Renström et al. (2023a) explored a *normative gender bias*. A normative gender bias is when a word (or word pair) is associated with individuals with normative cis-gender appearances. This was explored in three studies performed in both Swedish and English. In experiment 1, participants were shown a sentence composed of a target (e.g., *The person*) and a pronoun (*he/she* or *hen*) referring to the target. Participants were then asked to select a photo of the person they thought the sentence was about. Photos depicted not only cis-normative looking women and men but also more queer and non-normative looking individuals. The results showed that participants that had read the paired pronoun form *he/she* tended to select photos of normative looking individuals to a larger extent that non-normative looking individuals. In the *hen*-condition, there was no difference. That is, participants who read about a target person referred to as *hen*, tended to select photos of normative and non-normative looking individuals randomly. In a second experiment, the set-up was similar to the previously discussed experiment testing a male bias with the exception that the job now described a candidate for a position as a train attendant and that participants now could select photos of non-normative looking individuals. These results confirmed that *he/she* was associated with normative looking women and men, while *hen* was not. These results clearly indicate that paired forms, or feminization/balancing strategies make nonbinarity invisible. In support of this, Mirabella et al. (2024) show that in a grammatically gendered language (Italian), individuals with nonbinary gender identities report difficulties in expressing their identity, which could be because there is no readily available nonbinary pronoun (Koch and Lupyan, 2024). Similar results have been found for French (Knisely, 2020).

In a replication in English, the pronouns singular *they*, *ze,* and *he/she* were compared. The results showed that *he/she* was normatively biased as expected and that singular *they* was associated with normative-looking individuals. Hence, singular *they* is both male-biased and normatively biased. The neopronoun ze was not associated with any specific gender expression. Yet again, people may be unfamiliar with using *ze* as a nonbinary pronoun.

To date, the results paint a relatively complicated picture. The neopronoun *hen* in Swedish appears to broaden gender categories beyond the binary. However, in English, the story is more complicated. The most popular nonbinary pronoun, singular *they*, appears to be connected not only to masculinity but also to normativity. One reason may be that singular *they* has historical roots where its use has been generic, when gender is unknown or unimportant (Balhorn, 2004). As previously discussed, such neutrality is often associated with masculinity (Lindqvist et al., 2019; Bailey and LaFrance, 2017). While the neo- and nonbinary pronoun *ze* appears to perform better in that *ze* appears to include both women and non-normativity, there is still uncertainty about how readers perceive this word if they have no previous knowledge.

## Dual meaning of gender-inclusive pronouns

As previously mentioned, some gender-inclusive pronouns function in two ways. First, they can increase the visibility of gender identities outside the traditional binary categories when used as referents for a specific individual with a nonbinary gender identity—that is they are used to multi-gender. Second, gender-inclusive pronouns can be used generically, which instead decreases the influence and visibility of gender in language altogether—that is, they are used to de-gender. In this section, I will discuss attitudes and acceptability of the different meanings and how this dual meaning influences perceptions of the pronouns and conceptualization of gender.

### Attitudes and acceptance of different meanings of gender-inclusive pronouns

As mentioned, general attitudes toward the gender-inclusive pronoun *hen* in Swedish have become increasingly positive, or at least less negative, over 10 years (Gustafsson Sendén et al., 2015, 2021; Renström et al., 2024). General attitudes were the focus in these surveys, not attitudes toward different meanings.

In a study by Renström et al. (2022a), the authors assessed indirect attitudes toward *hen* in de-gendering and multi-gendering contexts by asking participants to rate sentences in both contexts. Participants were shown sentences with the paired pronoun *he/she* or *hen* in de-gendering contexts such as: “When a train attendant is sick, hen [he/she] should stay home,” or in a multi-gendering context referring to a specific individual: “Lex took a nap; she [he, hen] was very tired.” The participants rated the sentences on grammatical correctness, reading difficulty, and negative valence. The results showed that *hen* used in generic contexts (de-gendering) was more accepted, as shown in higher ratings on grammaticality and lower ratings of reading difficulty and negative valence, compared to *hen* in specific contexts (multi-gendering).

Similar findings are reported for singular *they* in English (Bradley et al., 2019; Bradley, 2020). Sentences with generic singular *they* were evaluated more positively (more grammatically correct and less offensive) compared to sentences with specific singular *they*. Similarly, Renström and Klysing (2024) found that singular *they* was more favorably evaluated in generic contexts than in specific contexts using a comparable sentence rating paradigm. Hekanaho (2020) found in a survey that attitudes toward nonbinary singular *they* were more positive than those toward other neopronouns, aligning with the findings of Renström et al. (2024).

However, many participants also disagreed with the nonbinary use of singular *they* as well, even when they accepted its use as a generic pronoun. One conclusion from this research was that many participants viewed gender as a binary construct, which led to their opposition to nonbinarity (Hekanaho, 2020).

Taken together, these studies indicate that people tend to have relatively positive attitudes about the use of gender-inclusive pronouns in generic, de-gendering contexts but not in nonbinary contexts.

To better gauge the acceptance of gender-inclusive pronouns, in another experiment (Renström et al., 2022a), participants were shown similar sentences as in the sentence rating paradigm described above, but with the pronoun missing and asked to fill in the missing word. The results showed that *hen* was overwhelmingly popular in generic contexts, even more so than the paired form *he/she*. In specific contexts, however, the participants preferred to assign a binary gender, even though the name used was neutral. The same result was found for singular *they* (Renström and Klysing, 2024): In generic contexts, singular *they* was preferred over binary pronouns, but in specific contexts, participants assigned a binary gender to the target by using *he* or *she*. These usage results align with what Hekanaho (2020) found, that singular *they* was more popular to use in generic contexts than binary gendered pronouns.

Regarding other pronouns, such as *ze*, there is less research to report. One reason is that *ze* is relatively unknown to English speakers in general (Hekanaho, 2020; Lindqvist et al., 2019; Renström et al., 2023a), which explains why some people may believe *ze* is misspelled *he* (Hekanaho, 2020). This implies that results from research using *ze* without specifying its use to the participants may suffer substantial measurement errors. Renström et al. (2024), using the same sentence rating paradigm and *ze* in generic and specific contexts, found that the nonbinary meaning of *ze* was seen as more grammatically correct than the generic meaning. There were no differences in reading difficulty and negative valence between using *ze* as a generic or a nonbinary pronoun. This could indicate that people interpret *ze* as a nonbinary pronoun designated for nonbinary people; hence, it should not be used generically. Hekanaho (2020) found that this is how participants reflected on *ze* in her study—*ze* should be seen as a gendered pronoun in a similar way that *he* and *she* is and hence is equally unsuitable to use generically, as, for instance, generic *he* is.

In sum, it seems that the generic meaning of gender-inclusive pronouns such as singular *they* and *hen,* are accepted, while the nonbinary meaning is still resisted. An empirical question is how these pronouns when specified in the different contexts influence gender conceptualizations.

To better understand how gender-inclusive pronouns in different meanings influence gender conceptualization, Renström et al. (2023a) performed the experiment described earlier where participants read about a candidate for a job position and were asked to select among a set of photos whom they thought was described in the text. This time, they added information about why the pronoun, in this case, *ze* and singular *they*, was used, thus eliminating ambiguous interpretations that may have been present in the earlier studies. That is, participants were in different conditions informed that the pronoun was used because the person had a nonbinary gender identity (multi-gendering), to anonymize (de-gendering), or they were not provided any information in a control condition. The results showed that when participants were explicitly informed that the pronoun was used because the referent had a nonbinary gender identity, the earlier normative bias was reversed. Participants tended to select a non-normative looking individual to a larger extent than a normative looking individual. This result was particularly strong for *ze* but, to a lesser degree, was also present for singular *they*. Interestingly, when participants did not receive any information in the control condition and were informed that the pronoun was used generically, the results were the same, showing a normative bias for both singular *they* and *ze*. This implies that when not informed about the use and when the context is ambiguous – that is, the pronoun could be used either to de-gender or to multi-gender, readers tend to infer a generic use, and this also leads to a normative bias.

Finally, because *ze* has been shown to be fairly unknown to the English-speaking population (Lindqvist et al., 2019), participants were asked if they had knowledge about the use of different pronouns as being nonbinary (Renström et al., 2023a). If people do not have knowledge about the use of a certain word, they will reasonably have problems implementing and interpreting the word. Because singular *they* was known to almost all participants (98%), analyses of differences in knowledge were not possible to run. However, for *ze*, knowledge about *ze* as a nonbinary pronoun dramatically decreased the normative bias, although there was still a tendency to select a normative-looking individual. In total, 204 participants had knowledge about *ze* (24% of the sample), meaning that this is quite a small sample to draw any definite conclusions from, but it appears that knowledge, at least when it comes to neopronouns, is conducive to its influence on gender conceptualization. However, an open question is why this knowledge appeared to be inconsequential for gender conceptualization for singular *they,* given that singular *they* was heavily associated with normative gender expressions even though the majority of the participants did know that *they* was a nonbinary pronoun (as well as a generic).

The greater acceptability of gender-inclusive pronouns’ use in generic compared to nonbinary contexts is likely rooted in the way that nonbinarity poses a challenge to cis-normativity (Morgenroth et al., 2020; Renström and Klysing, 2024). As mentioned, this duality may be related to different motives behind resistance against gender-inclusive pronouns (Renström et al., 2022a; Renström and Klysing, 2024), which I will discuss further in the next section.

## Politicization of gender and pronouns

Gender, along with other contentious issues such as the environment and race, has become increasingly politicized and, consequently, polarized. Consequently, gender-inclusive language has also been politicized. Politicization can be understood as a shift from discussing how things are to a debate driven by parties or partisans seeking to advance a political agenda. Mazzuca and Santarelli (2023) suggest this process can occur through various features. For instance, the framing of an issue—particularly the idea that things could be different—is a hallmark of politicization, though issues can also be reconstructed in the process.

A clear consequence of the politicization of the Swedish pronoun *hen* is that when *hen* was first introduced, one of the largest newspapers in Sweden forbid its journalists from using *hen* as it would send signals about political standings.

Moreover, the Language Council of Sweden, providing unofficial recommendations on language use, recommended that *hen* be avoided because it was so highly politically charged.^2^ Even though this recommendation was later revoked, the need for caution in using *hen* was still emphasized. In line with this, extensive research shows that gender-inclusive language reforms, including the use of gender-inclusive pronouns, face resistance (Blaubergs, 1980; Parks and Roberton, 1998; Bradley, 2020; Bradley et al., 2019; Hekanaho, 2020; Vergoossen et al., 2020a).

This resistance appears largely grounded in gender-ideological convictions about gender being an essential and binary category and a desire to keep gendered power structures intact (Douglas and Sutton, 2014; Parks and Roberton, 2005). Yet, some people oppose such reforms based on more linguistic reasons, such as preferring the linguistic status quo (Vergoossen et al., 2020a) or so-called linguistic prescriptivism (Bradley, 2020). However, according to Hekanaho (2020), such arguments about linguistic awkwardness may reflect a preference for a more socially accepted opposition but be rooted in discomfort with nonbinarity.

In a qualitative analysis of arguments against the use of Swedish *hen*, Vergoossen et al. (2020a) found four dimensions of resistance. Using earlier taxonomies of critical arguments against gender-fair language reforms in the past (i.e., Blaubergs, 1980; Parks and Roberton, 1998), they found that roughly 80% of the arguments against the use of *hen* could be coded into the previously existing categories. Hence, while *hen* is a new word with new implications, the arguments against its use were the same as the arguments against the use of the paired form *he/she* when it was suggested to be used instead of a generic *he,* and these arguments have been consistent over almost 50 years. Two categories of arguments were new and specifically related to *hen* as a gender-inclusive pronoun: gender-inclusive pronouns distract communication, and gender information about a target person is important. Based on the coded categories, four overarching dimensions captured assumptions and beliefs underlying criticism against gender-inclusive or gender-fair language reforms. The dimension with the most arguments (ca. 40%) was a defense of the linguistic status quo, encompassing arguments mainly relating to that change is too difficult or unnecessary. The second dimension was related to the fact that sexism and cisgenderism are acceptable (encompassing ca. 30% of the arguments). Here, arguments about binary biology and hostility against people with nonbinary gender identities were dominant. The third dimension, diminishing of the issue and its proponents (27%), contained disparaging reactions to both gender-inclusive language and people who advocate for it. Hostile, ridiculing, and denigrating comments were common. This dimension is also closely connected to the nonbinary use of *hen*, which is what the arguments were about. Finally, some people claimed they did not want to use *hen* because they perceived it to be distracting in communication (6%). Although relatively small, this latter dimension indicates the political nature of gender-inclusive pronoun use (Vergoossen et al., 2020a).

Taken together, most of the categories of arguments were related to the nonbinary use of *hen*. But, what predicts these negative attitudes? In their general attitude surveys, Gustafsson Sendén et al. (2015, 2021) and Lindqvist et al. (2016) found that some predictors appear relatively stable. For instance, individuals with a more left-leaning ideology, those identifying as women or nonbinary, younger people, and those with a general interest in gender issues tend to hold more positive attitudes toward *hen*.

However, as de-gendering and multi-gendering strategies to some extent challenge core convictions of different ideologies, the origins of resistance against gender-inclusive pronouns may also differ depending on which strategy is salient (Morgenroth et al., 2020; Renström et al., 2022a), necessitating further scrutiny of different predictors for different meanings of gender-inclusive pronouns.

### Ideological origins of resistance

De-gendering strategies aim to remove or minimize the salience of gender altogether, while multi-gendering strategies aim to draw attention to the fact that gender is not binary (Morgenroth and Ryan, 2020). The duality of gender-inclusive pronouns may be related to different origins of resistance (Renström et al., 2022a). Because de-gendering strategies remove gender cues, it may lead individuals to not think about gender and, therefore, not question its binary nature (Morgenroth et al., 2020). In relation, singular *they*, used in a de-gendering way, is associated with normative gender expressions even though most participants reported that they were knowledgeable about singular *they* as a nonbinary pronoun (Renström et al., 2023a).

The use and perception of gender-inclusive language reforms are not just a matter of personal preference or identity. They also relate to the motivation to defend a hierarchical and traditional binary gender system as expressed in language. Social dominance orientation (SDO) and right-wing authoritarianism (RWA) are two status-legitimizing ideologies, meaning that they entail the endorsement of worldviews where social inequalities between groups are seen as legitimate (Major and Kaiser, 2017). Both SDO and RWA predict gendered prejudice, including sexism (Duckitt and Sibley, 2010; Van Assche et al., 2019) and homophobia (Crawford et al., 2016). It therefore appears plausible that both SDO and RWA might predict negativity to gender-inclusive pronouns through their role in challenging conservative beliefs about group hierarchies. However, there are important differences between SDO and RWA that may influence resistance against gender-inclusive pronouns differently depending on the active strategy.

Social Dominance Orientation expresses the motivational goal for group-based dominance and superiority (Duckitt, 2001; Pratto et al., 1994; Sidanius and Pratto, 1999; Sidanius et al., 2004). People high in SDO see the world as a competitive jungle, which entails a struggle for resources (Duckitt et al., 2002)—a zero-sum game. In terms of gender relations, this means a belief that if sexual minority groups gain status and privileges, the higher-status gender groups lose out, making people high in SDO motivated to counteract minority rights progress (Poteat and Mereish, 2012). Individuals high in SDO should be primarily concerned with keeping a linguistic structure highlighting the order and construction of gender hierarchies in society, such as using the paired pronoun form *he/she*. Moreover, this pronoun use also positions men as the dominant group, which could explain why men often are more negative toward gender-inclusive language that diminishes group saliency than women (Douglas and Sutton, 2014; Lindqvist et al., 2016). As de-gendering strategies function to remove gender cues, individuals high in SDO may be more skeptical of such procedures and thus particularly dislike the use of singular *they* in de-gendering contexts. Individuals high in SDO might not be overly concerned about singular *they* in multi-gendering contexts because this highlights a “third” gender group in society that the ingroup can dominate, be it men or women. In a study using the sentence rating paradigm and the fill-in-the-blanks task described earlier, Renström and Klysing (2024) found that individuals high in SDO were less positive when evaluating sentences with singular *they* in a de-gendered context, but there was no effect of SDO on evaluations of sentences in the multi-gendered contexts. This could reflect that individuals who prefer a clear gender structure and hierarchy do not mind an extra gender group since that group will be a minority group and, hence, a group that could be dominated. Recently, the triple form *he/she/they* has started to emerge. It is possible that individuals high in SDO might prefer this form even more than *he/she* since it positions a “third” group. Additionally, given the importance of ordering—such as presenting *he* before *she*—the triple form might be more appealing to women with high levels of SDO.

Right-wing authoritarianism (RWA) is an ideologically based personality feature where individuals who are high in RWA desire tradition and conformity to conventional ways and are characterized by an emphasis on submission to authority and upholding norms of social order (Altemeyer, 1981). Therefore, a high level of RWA leads to dislike of individuals who violate these norms (Peresman et al., 2021). Individuals high in RWA perceive norm-violating social groups, such as gender non-conforming people, as threats to the ingroup and, therefore, become motivated to protect the ingroup against these threats (Renström et al., 2022b; Sidanius et al., 2004). One expression of a traditional and conservative belief system is the view of gender as an essential and binary category (Hyde et al., 2019; Tee and Hegarty, 2006).

The multi-gendering strategy challenges these traditional gender roles and norms by implying that there are more than two genders, thereby questioning the validity of binary gender as a system of societal organization (Morgenroth and Ryan, 2020). For instance, gender essentialism has been linked to prejudice against individuals who violate gender norms, such as women in leadership positions (Skewes et al., 2018). Similarly, binary views of gender have been associated with increased prejudice toward nonbinary individuals (Morgenroth et al., 2020).

In the context of singular *they*, transcendent views of gender (i.e., lower endorsement of strict gender roles) were positively associated with attitudes toward singular *they* when used in a nonbinary, multi-gendering context but not when used in a de-gendering context (Bradley et al., 2019). This indicates that traditional views and beliefs about gender as binary are more strongly tied to the understanding of gender-inclusive pronouns in nonbinary contexts. As a general preference for traditions and norms, RWA correlates with the endorsement of traditional gender roles (Peterson and Zurbriggen, 2010). Dislike of gender norm violators (Perez-Arche and Miller, 2021) should, therefore, lead to stronger resistance to the multi-gendered strategy due to it questioning the validity of a traditional, binary gender system.

Renström and Klysing (2024) found that RWA predicted negativity toward singular *they* in the multi-gendered contexts. In Study 1, people high in RWA were less likely to use singular *they* in multi-gendered contexts. In Study 2, RWA predicted negative evaluations of singular *they* in the multi-gendered contexts but less so in de-gendered contexts. RWA encompasses views of minorities as threatening to the majority’s conventional way of life (Renström et al., 2022b; Sidanius et al., 2004). Since highlighting the existence of other gender identities than the binary woman/man might constitute such a threat, individuals high in RWA should be more negative toward such practices.

Taken together, these results point to the importance of considering the different contexts or meanings that some gender-inclusive pronouns have to better understand resistance against them and why this may vary.

## Challenges and future directions

Pronouns are considered a closed word class that rarely changes, but recently, several languages have implemented additional third-person pronouns. Pronouns have become important identity markers and have consequences for how individuals relate to themselves and others, meaning we face a unique situation. Researchers should take the opportunity to follow the implementation of gender-inclusive pronouns in other languages, as this is a rare opportunity. In this review, I have tried to cover some aspects of gender-inclusive pronouns that are important to consider in this line of research.

First, when researching nonbinary pronouns specifically, one must consider the general knowledge of the pronoun. For instance, English *ze* is relatively unknown, which influences how results using this pronoun should be interpreted and is likely to lead to large measurement errors and, potentially null effects. In addition, a recent study on Dutch found that half of the sample was not familiar with gender-inclusive pronouns in Dutch (DeCock et al., 2024), indicating problems in measuring attitudes.

Second, the dual meaning of some gender-inclusive pronouns, which are well-known, also runs the risk of compromising interpretation and introducing measurement error if the research is not designed with this dual meaning in mind. Given that different ideological convictions may underlie resistance against using gender-inclusive pronouns in different contexts (i.e., corresponding to the de-gendering and multi-gendering strategies), failure to include this as a factor will lead to difficulties in drawing conclusions.

Relatedly, but also separate from the dual meaning and the underlying ideological resistance, is the politicization of gender. When gender-inclusive language, including pronouns, becomes a political position statement, the study of their effects on gender conceptualization also becomes compromised. This polarization is unlikely to recede over time. New research shows that younger men perceive gender equality as a threat to a larger extent than older men (Off et al., 2022). Moreover, the recent uprise of the misogynistic online milieu, referred to as the manosphere and its influencers, mainly addresses young men who are more susceptible to its anti-feminist messages than older men (Renström and Bäck, 2024).

Moreover, such misogynistic communities often intersect with alt-right communities, both of which promote traditional gender roles and nuclear families as societal ideals. Gender-inclusive language, which aims to increase the visibility of sexual minorities and reduce gender biases, directly challenges these ideals and is thus met with resistance. However, their implementation faces significant challenges if resistance persists and political polarization intensifies. Currently, there is very little research in this area, and a deeper understanding is needed of how the politicization of gender shapes attitudes toward, the use of, and the effects of gender-inclusive pronouns.

To date, there is also limited research on pronouns in other languages published in English. Hence, cross-cultural studies that explore gender-inclusive pronouns in different languages using similar study setups are desired. In such research, it is also desirable to include languages that vary in grammatical gender. As mentioned, gender-inclusive language may be more or less difficult to implement depending on grammatical structure. Regarding pronouns specifically, there is a lack of research on grammatical gendered languages. However, a recent Italian study showed that nonbinary individuals struggled to express their identity linguistically (Mirabella et al., 2024), which could be a consequence of the grammatical gender structure of Italian. Such research highlights the need for further scrutiny of pronouns in different languages.

Moreover, the present review is mainly concerned with Swedish and English, and while I call for more research on other Western languages with a variation of grammatical gender, a global perspective is also desirable. This would provide both a new linguistic and cultural/political perspective. For instance, there is no differentiation between feminine and masculine pronouns in spoken Chinese, but there is in written Chinese.

### Practice implications

As should be evident, words and word choices matter. But how should this be translated into practice when the concept of gender is so contentious that use of the associated words has become political position statements? This question has no straightforward answer, and more research is required. What can be stated is that, despite the contentious nature of gender issues, gender-majority participants are not overwhelmingly negative toward gender-inclusive pronouns (e.g., Renström et al., 2022a, 2022b; Bradley, 2020). Furthermore, as shown by eye-tracking studies, gender-inclusive pronouns in texts are not particularly difficult to process (Vergoossen et al., 2020b).

In terms of gender-fair language, an important finding is that multi-gender strategies in equal opportunity statements within organizational descriptions did not reduce gender-majority participants’ organizational attraction (Klysing et al., 2021).

However, in languages such as German, gender-inclusive language—particularly paired forms—was associated with reduced comprehensibility (Friedrich and Heise, 2019).

These results suggest that while some individuals may resist gender-inclusive language, its effects are not insurmountable. A practice recommendation, therefore, is to adopt gender-inclusive language whenever possible. This recommendation is further supported by research indicating that exposure to gender-fair language in languages such as Norwegian and German increases subsequent usage of such language (Kuhn, 2021).

## Footnotes

^1^ https://www.oed.com/discover/a-brief-history-of-singular-they?tl=true

^2^The language council of Sweden is the official organ in Sweden that deals with language care and language politics. It is part of the Institute for language and folk lore, https://www.isof.se/other-languages/english/about-the-institute.
